# Fully Coupled Model for Frequency Response Simulation of Miniaturized Cantilever-Based Photoacoustic Gas Sensors

**DOI:** 10.3390/s19214772

**Published:** 2019-11-02

**Authors:** Sheng Zhou

**Affiliations:** 1 Department of Physics and Astronomy, VU University Amsterdam, 1081 HV Amsterdam, The Netherlands; s.zhou@vu.nl; 2 LaserLaB Amsterdam, VU University Amsterdam, 1081 HV Amsterdam, The Netherlands

**Keywords:** photoacoustic spectroscopy, viscous damping, sensor miniaturization, COMSOL

## Abstract

To support the development of miniaturized photoacoustic gas sensors, a fully coupled finite element model for a frequency response simulation of cantilever-based photoacoustic gas sensors is introduced in this paper. The model covers the whole photoacoustic process from radiation absorption to pressure transducer vibration, and considers viscous damping loss. After validation with experimental data, the model was further applied to evaluate the possibility of further optimization and miniaturization of a previously reported sensor design.

## 1. Introduction

Photoacoustic (PA) spectroscopy has been recognized as a sensitive trace gas detection technique [1]. In a typical PA system, a modulated light beam with a proper wavelength is sent into a gas sample cell where target gas molecules are excited by the beam. A pressure wave is produced by the periodic thermal expansion of the gas sample results from collisional relaxation of excited gas molecules and is detected by various pressure transducers. The detected pressure wave amplitude is used to evaluate the concentration of target gas in the sample. Commonly, the acoustic resonance of the gas column in the cell is used to improve the PA signal and a commercial microphone mounted on the gas cell is used to detect the pressure wave. Finite element methods (FEM) have been successfully implemented to evaluate the eigen frequencies, modes, and frequency response of PA cells with complex geometries for cell design optimization, as analytical solutions are restricted to simple cell geometries [2,3,4]. Furthermore, it has been demonstrated that FEM can even quantitatively simulate the PA signal [5]. Most FEM models, however, do not include the pressure transducer in the model. This is feasible when commercial microphones, which normally have a flat frequency response under 20 KHz, were used as the pressure transducer. Those microphones can be regarded as part of the rigid wall of the PA cell when the PA signal frequency is much lower than 20 KHz. However, with the development of a new branch of PA systems that utilize the mechanical resonance of the pressure transducers for PA signal improvement, it becomes essential to include the pressure transducer in the FEM model and consider the acoustic-vibration coupling to simulate the PA system properly [6,7]. Additionally, with the continuous development of miniaturized PA sensors, it is important to include the viscous damping in the FEM model. In fact, viscous damping may play a dominant role on PA signal reduction when the viscous penetration depth is at the same order of the gas cell dimensions [8,9].

Recently, we introduced a series of cantilever-based miniaturized PA sensors to the field in which customized micro-cantilevers were used as the pressure transducer and glass tubes with an inner diameter as small as 0.6 mm was used as gas cells [10,11,12]. To guide the development of such gas sensors towards further optimization and miniaturization, a fully coupled sensor model based on Comsol Multiphysics is proposed in this paper. The model covers the whole photoacoustic process from radiation absorption to pressure wave generation, and then to transducer vibration. Viscous damping was included as an energy loss mechanism. This model is applied to a PA sensor reported in [12], showing that it can match the experimental data on the sensor frequency response quantitatively well. Simulations of how different sensor parameters were going to influence the PA signal are further given, suggesting the possibilities and limitations of miniaturization and signal enhancement for cantilever-based PA sensors.

## 2. Experimental Setup

**Figure 1:**
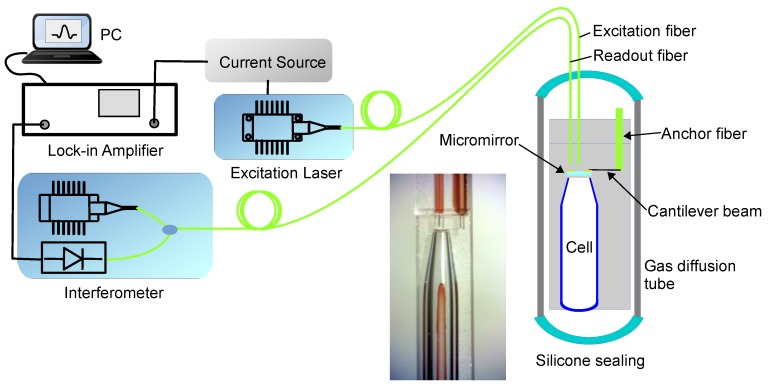
Experimental setup.

The simulation model is based on an experimental setup as schematically shown in Figure 1, which was described in detail in [12]. The PA sensor is shown on the right part of the figure. It mainly consists of an one end sealed transparent glass tube as the cell and a cantilever beam hanging a micromirror over the cell inlet as the pressure transducer. The micromirror was made from a piece of square glass plate (width = 300 μm, thickness = 30 μm), and was made transparent at the center and reflective elsewhere. Two fibres were aligned towards the micromirror, of which the excitation fiber transmits the excitation laser beam into the cell through the transparent region of the micromirror, while the readout fiber points towards the reflective region so that an interferometer (OP1550, OPTICS11) it is connected to can read the micromirror deflection signal. When the wavelength of the excitation laser (Oclaro, TL5000VCJ) is modulated around one of the target gas’ absorption line (1530.37 nm for Acetylene in this case) by a current source, gas molecules inside the cell are heated up periodically and hence generate a pressure wave that vibrates the micromirror. A lock in amplifier (SRS865, SRS) connected to the interferometer was used to extract the micromirror vibration amplitude signal at 2nd harmonica of the excitation frequency. All components of the PA sensor were enclosed in a gas permeation tube because the sensor was designed to be immersed in transformer oil for dissolved $C_{2}H_{2}$ detection in our experiments. A photo of the inner structure of the sensor part in the setup was included in the figure to indicate the scale (optical fibres in the figure have a diameter of 0.125 mm). To be able to compare the real sensor performance with the simulation model described hereafter, the sensor’s frequency response was collected when it was immersed in an oil standard sample (True North) with a certificated dissolved $C_{2}H_{2}$ concentration (10(1±10%) ppm). During data collection, the central excitation wavelength was fixed at the $C_{2}H_{2}$ absorption line and the excitation frequency was swept around half the resonance frequency of the transducer.

## 3. Modeling of the PA Sensor

**Figure 2:**
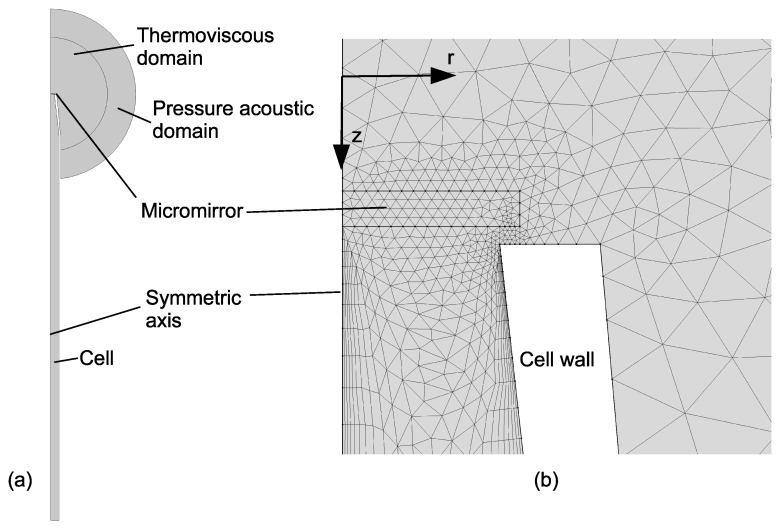
Model of the PA (photoacoustic) sensor (**a**) and mesh at the micromirror region (**b**).

A sketch of the PA sensor modeled in Comsol Multiphysics is shown in Figure 2a. To reduce the computational cost, a 2D axisymmetric model was used. The cantilever pressure transducer was simplified with a round silica glass micromirror (diameter of 300 μm, thickness of 30 μm) combined with a spring foundation constraint that was applied to it. Ideally, the spring constant of the spring foundation should be set to the same as that of the cantilever beam of the pressure transducer. However, because the mechanical properties of the cantilever beam were unknown in our experiments, the spring constant was chosen to ensure that the resonance frequency value resolved by the model overlaps with that of the experiment’s result. The permeation tube of the sensor was also not included in the model for simplicity. Key parameters of the experimental setup that were directly implemented in the model are listed in Table 1.

In order to get a correct assessment of the damping loss around the micromirror and the cell inner wall, and to include the radiation absorption process directly into the model, the gas in the gas cell and around the cell inlet was modeled with a thermoviscous interface by simply using the material properties of standard air defined in the Comsol library. Because of the viscous property and much higher thermal conductivity of the cell walls, they were treated as no-slip and isothermal boundaries. A pressure acoustics layer was used to truncate the computational domain. The cell wall in the pressure acoustic domain was set as a sound hard boundary. The outer spherical perimeter of the pressure acoustic domain was set as spherical wave radiation condition so that acoustic wave experiences little reflection on the perimeter. Other parameters, such as radius of domains and maximum mesh element size, were chosen to ensure the convergence of the simulation results (e.g., parameter independent frequency response, and relative error estimations smaller than 0.005), as well as reasonable computational time.

The energy transfer from the excitation laser beam to the gas molecules in the cell was modeled using a heat source condition in the air domain inside the cell, with heat power density calculated below.

Assuming that the transparent window at the center of the micromirror does not affect the excitation laser beam propagation, the beam divergence from the excitation fiber tip can be treated as a Gaussian beam with an initial beam diameter (full $1/e^{2}$ width), $2w_{0}$, equal to the mode-field diameter of the fiber ( 10.5 μm). The beam radius at a position *z* (set *z* = 0 at the excitation fiber tip surface) can be described as:

(1) $$w=w_{0}\sqrt{1+{\left(\frac{\lambda z}{\pi w_{0}^{2}}\right)}^{2}}$$

with λ being the wavelength. The light intensity of the beam can be then described as:

(2) $$I\left(r,z\right)=I_{0}\exp\left(-\frac{2r^{2}}{w^{2}}\right)$$

(3) $$I_{0}=kP\exp\left(\frac{2}{\pi w^{2}}\right)$$

where $I_{0}$ represents the on-axis light intensity, *P* is the power of the excitation laser, and *r* is defined as the distance to the beam axis. *k* is the transmission coefficient of the excitation laser beam from the laser to the cell, which considers the coupling loss between fiber connectors and power loss due to micromirror reflection, absorption and scattering. The heat power density at any location in the domain, which defines the heat source input in the model, is finally calculated as [6]:

(4) H(r,z)=αI(r,z)

where α represent the absorption coefficient of the target gas in the cell. According to the calibration result detailed in [12], a dissolved $C_{2}H_{2}$ concentration of 10 ppm in the transformer oil corresponds to a gas phase $C_{2}H_{2}$ concentration of 8 ppm in the gas cell. This leads to an absorption coefficient of 9.26e−4 m^−1^, calculated at atmosphere pressure and room temperature, based on the HITRAN database [13].

The mesh around the micromirror region is presented in Figure 2b. To properly resolve the excitation laser beam, boundary layer mesh elements were applied to the region around the cell axis, with a first layer thickness of $w_{0}/2$. Similarly, to include the viscous damping properly, boundary layer mesh elements were applied to the air close to the cell wall with a first layer thickness of dvisc/5 and edge mesh elements were applied to the cell inlet to a micromirror gap region with a maximum element size of min(dvisc/3,gap/3), where dvisc is the viscous penetration depth [14], defined as:

(5) $$dvisc=\sqrt{\frac{\mu }{\pi \rho f}}$$

with μ as the dynamic viscosity, ρ as the static density, and *f* as the PA signal frequency.

## 4. Results

**Figure 3:**
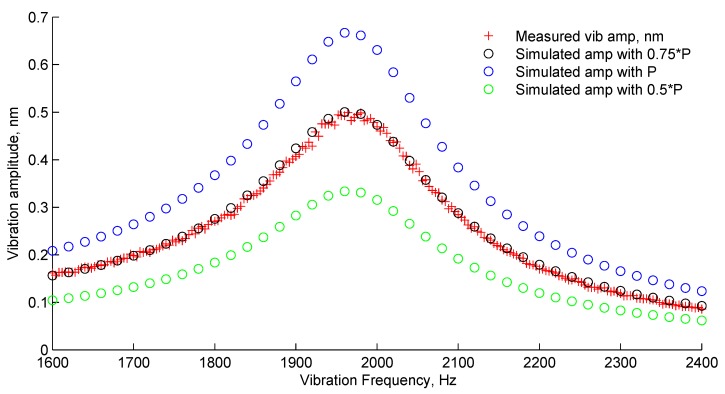
Experimental and simulated sensor frequency response.

**Figure A1:**
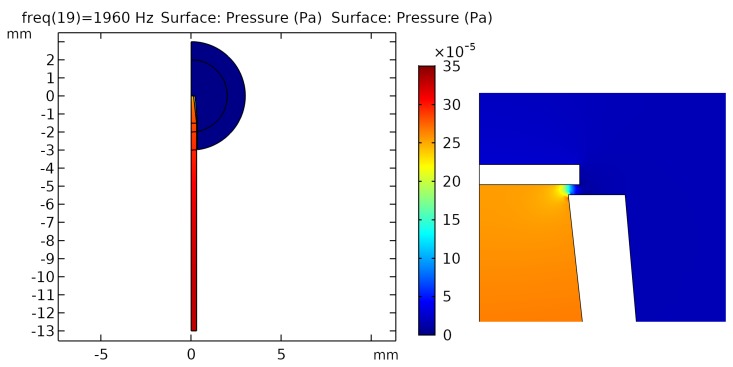
The left figure shows the simulated pressure profile of the sensor cell at resonance frequency. The right figure shows an enlarged view of the cell gap region.

The model was run to evaluate the micromirror vibration amplitude as a function of the heat power density modulation frequency. The simulated pressure profile at the resonance frequency is included in Figure A1. The simulated vibration amplitude of the micromirror central point around the first Eigen mode is compared to that measured in the experiment, as shown in Figure 3. From the frequency response curves, one can calculate the quality factor of the system [15]. The quality factor was calculated to be 10 for both experimental and simulation results and is independent from the transmission coefficient value set in the model. This indicates that the model can simulate the viscous damping that occurred in the sensor very well. Moreover, when *k* was set to 0.75, the simulated frequency response of the sensor almost overlapped with the experimental one. When *k* was varied between 0.5 and 1, the simulated frequency response was at the same order as that measured with the experiment. This result shows that the proposed model could simulate the frequency response of real PA gas sensor qualitatively well.

**Figure 4:**
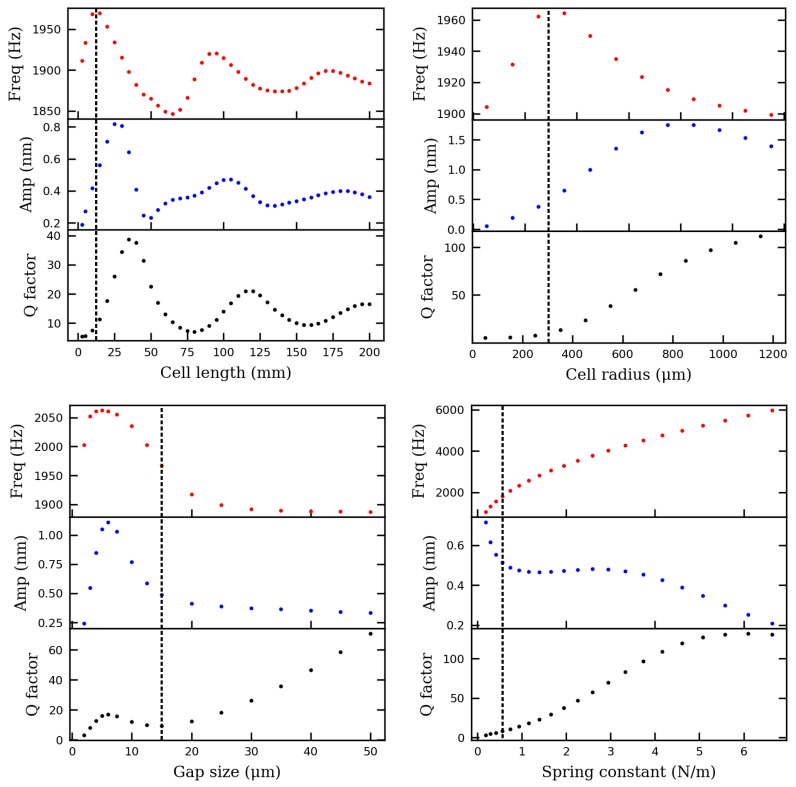
Influences of geometrical parameters to resonance frequency, cantilever vibration amplitude, and quality factor of the PA sensor. Dashed lines indicate original sensor parameters.

It is well known that, distinguished from conventional absorption spectroscopy, the signal in photoacoustic spectroscopy is independent from the cell length and becomes higher when the cell radius is reduced [16]. With increasing interests developed around photonic chip based gas sensors [17,18,19,20], it would be interesting to evaluate the miniaturization potential of cantilever-based PA sensors, as a guidance for the possible photonic integration of photoacoustic gas sensors in the future. Parametric sweeps based on the above model were carried out for this purpose. Single parameters including cell length, cell inner radius, cell inlet to micromirror gap size, and cantilever spring constant were swept around their designed values, and the sensor frequency response was simulated and collected. Three parameters, including resonance frequency, micromirror vibration amplitude at resonance, and quality factor, were further extracted from the simulated frequency response and plotted in Figure 4.

To calculate the quality factors, simulated frequency response curves were fitted to a displacement amplitude function of forced oscillation [15]:

(6) $$A=\frac{b}{\sqrt{{\left(f^{2}-f_{0}^{2}\right)}^{2}+{\left(rf\right)}^{2}}}$$

where $f_{0}$, *r*, *b* are the fitting parameters. The fitting reached a relative residual smaller than 10% for all points. The quality factor is then calculated by:

(7) $$Q=\frac{f_{0}}{r}$$

As seen in Figure 4, the oscillation behavior of the frequency response while sweeping the cell length suggests some complex coupling between the gas volume and the cantilever. Assuming an acoustic velocity of 340 m/s, the acoustic wavelength (λ) at 1940 Hz is about 176 mm, which is approximately two times the period of the oscillations. However, according to this simulation, the cell length at around 25 mm leads to the highest PA signal, even though it is much shorter than λ/4. There is a proper hypothesis for this behavior.

Firstly, due to the diameter shrinkage at the cell outlet region and/or the influence of the air gap between the micromirror and the cell outlet, a cell with a length of 25 mm acts equivalent to a λ/4 long tube resonator with one end closed, which has a fundamental acoustic resonance of around 1940 Hz.

Secondly, when increasing the cell length from 25 mm, every increment of cell length by λ/2 creates a new match of the cantilever mechanical resonance with a higher acoustic resonance mode of the tube resonator, which leads to a new vibrational amplitude peak.

**Figure A2:**
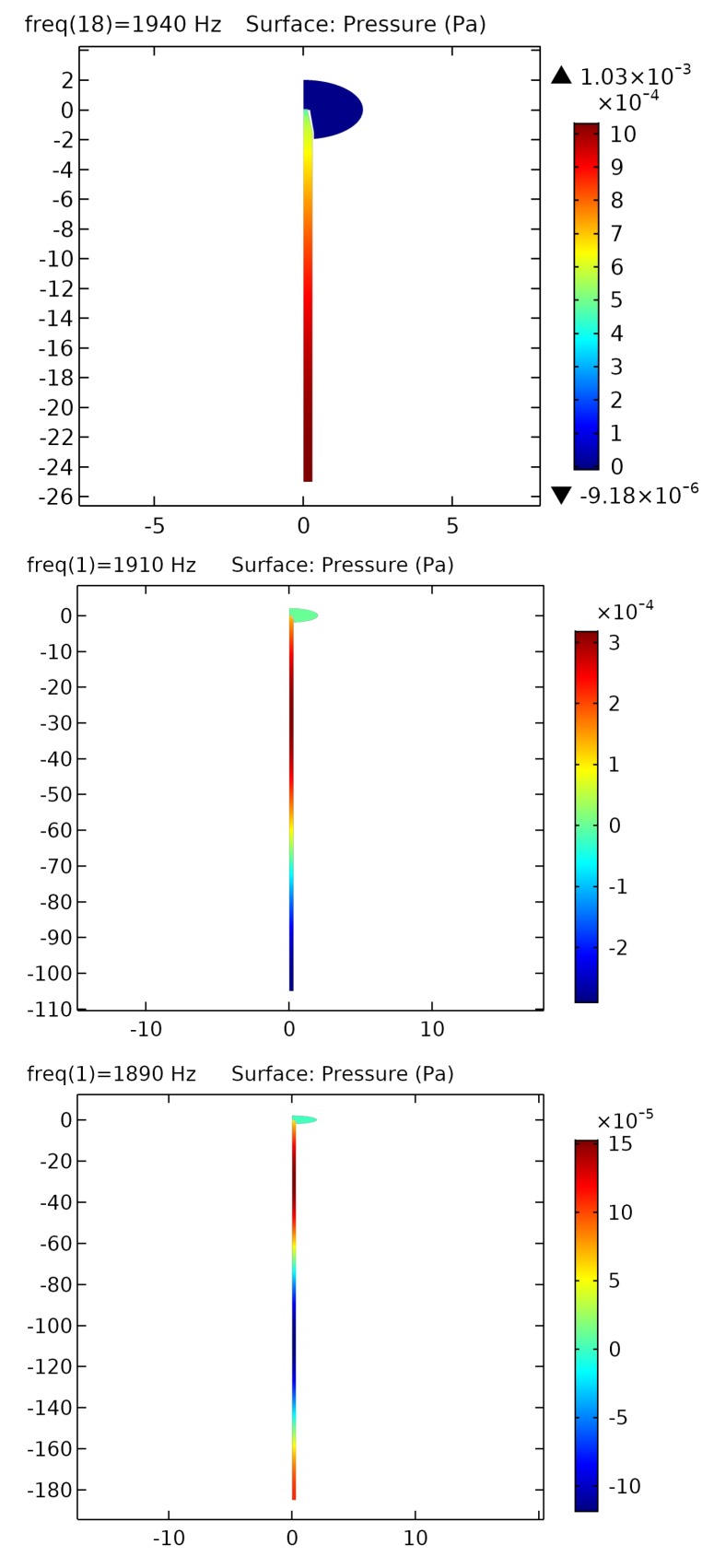
Pressure profile in the cell at resonance frequencies when the cell length is set to 25 mm, 105 mm, and 185 mm respectively. The x axis of each graph is manually scaled for visibility.

This hypothesis is partially verified by the pressure profile data captured while the cell length was set to 25 mm, 105 mm, and 185 mm respectively, as shown in Figure A2. Pressure standing waves are clearly visible at those cell lengths.

While sweeping the spring constant, the vibration amplitude at resonance ($f=f_{0}$) doesn’t follow the inverse of frequency (1/f) accordingly as defined by Equation (6). This could be explained by a match between the acoustic resonance of the air volume and the mechanical resonance of cantilever, when the spring constant is around 3 N/m.

Furthermore, increasing the cell radius could increase the PA signal most significantly, which shows that the cell diameter in the current sensor design is not optimal. The model predicts that the PA signal increased by two times if the cell radius was changed from 0.3 mm to 0.8 mm, while other parameters are fixed.

The modeling results for gap size sweep indicates that when the gap size was reduced from 15 μm to 5 μm, the PA signal doubled, which is likely due to better coupling between the pressure wave and the pressure transducer. A further reduction of the gap size reduced the PA signal a lot, most likely due to the so-called "breathing effect" of the narrow gap region [21].

## 5. Conclusions and Discussion

In conclusion, a fully coupled cantilever-based PA sensor model was introduced in this paper. It was applied to a miniaturized PA sensor reported in [12], showing that it could match experimental results quantitatively well.

The model was further applied to investigate how different sensor parameters were going to influence the sensor performance. According to the parametric sweep results, it seems that the signal to noise ratio of the sensor reported in [12] could be further improved by reducing the gap size, considering environmental acoustic noise as the dominant noise source [11]. Moreover, it seems that matching the acoustic and mechanical resonances of the PA cell and the pressure transducer could be an effective signal enhancement method that is worth further investigation. Furthermore, as seen from the results, the cell radius and the cell length were already limiting the sensing performance of the current sensor design. Further miniaturization is expected to increase the influence of viscous damping on the cell wall and hence deteriorate the gas sensing performance even further. Based on the modeling results, a semi photonic integration approach, where all components other than the gas cell are integrated into a photonic circuit, might combine the high sensitivity of photoacoustic spectroscopy with the mass production potential of photonic chips in future.

## Figures and Tables

**Table 1:** Parameters used for the model.

Parameter	Value	Description
P	24	Excitation laser power (mW)
t1	30	Micromirror thickness (μm)
r1	300	Cell main body inner radius (μm)
l1	13	Cell length (mm)
r2	133	Cell inlet inner radius (μm)
t2	50	Cell wall thickness (μm)
g	15	Cell inlet to micromirror gap size (μm)
l2	1.5	Cell inlet section length (mm)
